# Supplementary material for: Anti-CD44 Variant 10 Monoclonal Antibody Exerts Antitumor Activity in Mouse Xenograft Models of Oral Squamous Cell Carcinomas
Source: Int J Mol Sci. 2024 Aug 24;25(17):9190. doi: 10.3390/ijms25179190 (PMC11395228; doi:10.3390/ijms25179190)
Supplement: Supplementary file 1 [file ijms-25-09190-s001.zip › ijms-3164526-supplementary.pdf]

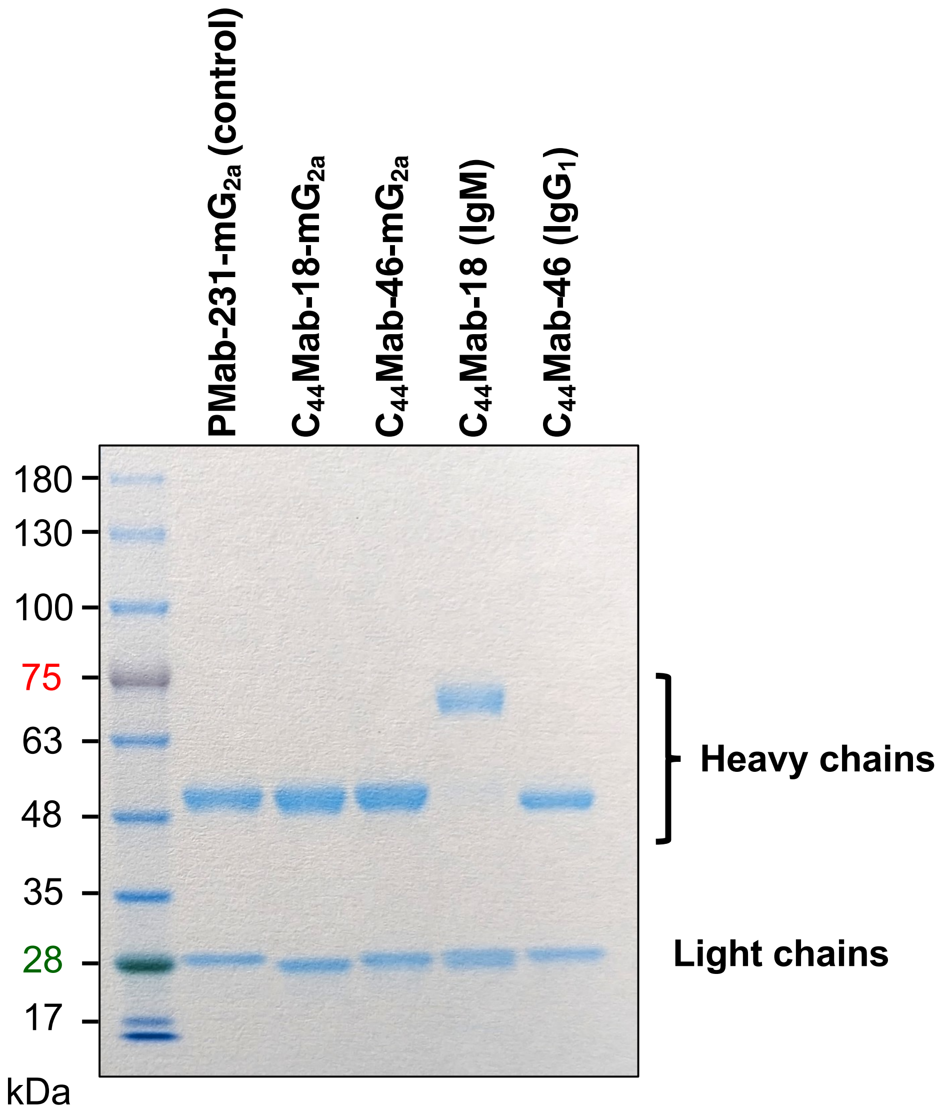

Supplementary Figure S1. Confirmation of the purified mAbs. MAbs (2 μg) were treated with sodium dodecyl sulfate (SDS) sample buffer containing 2-mercaptoethanol (Nacalai Tesque, Inc.). Proteins were separated on 5%–20% polyacrylamide gel (FUJIFILM Wako). The gel was stained by Bio-Safe CBB G-250 Stain (Bio-Rad Laboratories, Inc.).

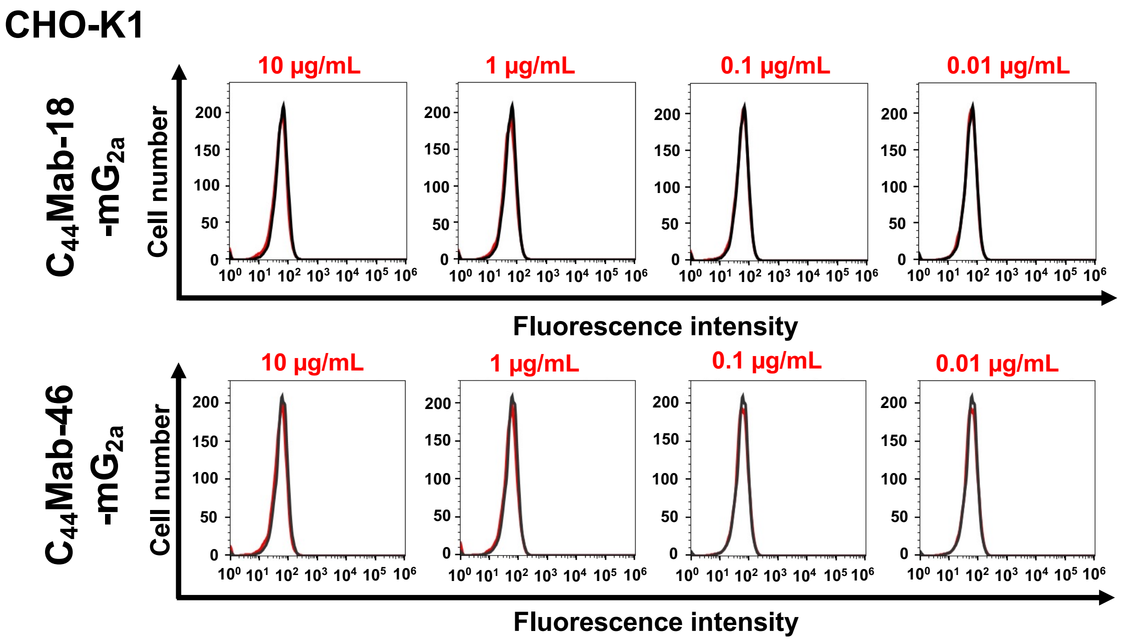

Supplementary Figure S2. Flow cytometry using C_{44}Mab-18-mG_{2a} and C_{44}Mab-46-mG_{2a} against CHO-K1 cells. CHO-K1 cells were treated with buffer control (black) or 10-0.01 µg/mL of C_{44}Mab-18-mG_{2a} and C_{44}Mab-46-mG_{2a} (red). The cells were further treated with Alexa Fluor 488-conjugated anti-mouse IgG. Fluorescence data were analyzed using the SA3800 Cell Analyzer.

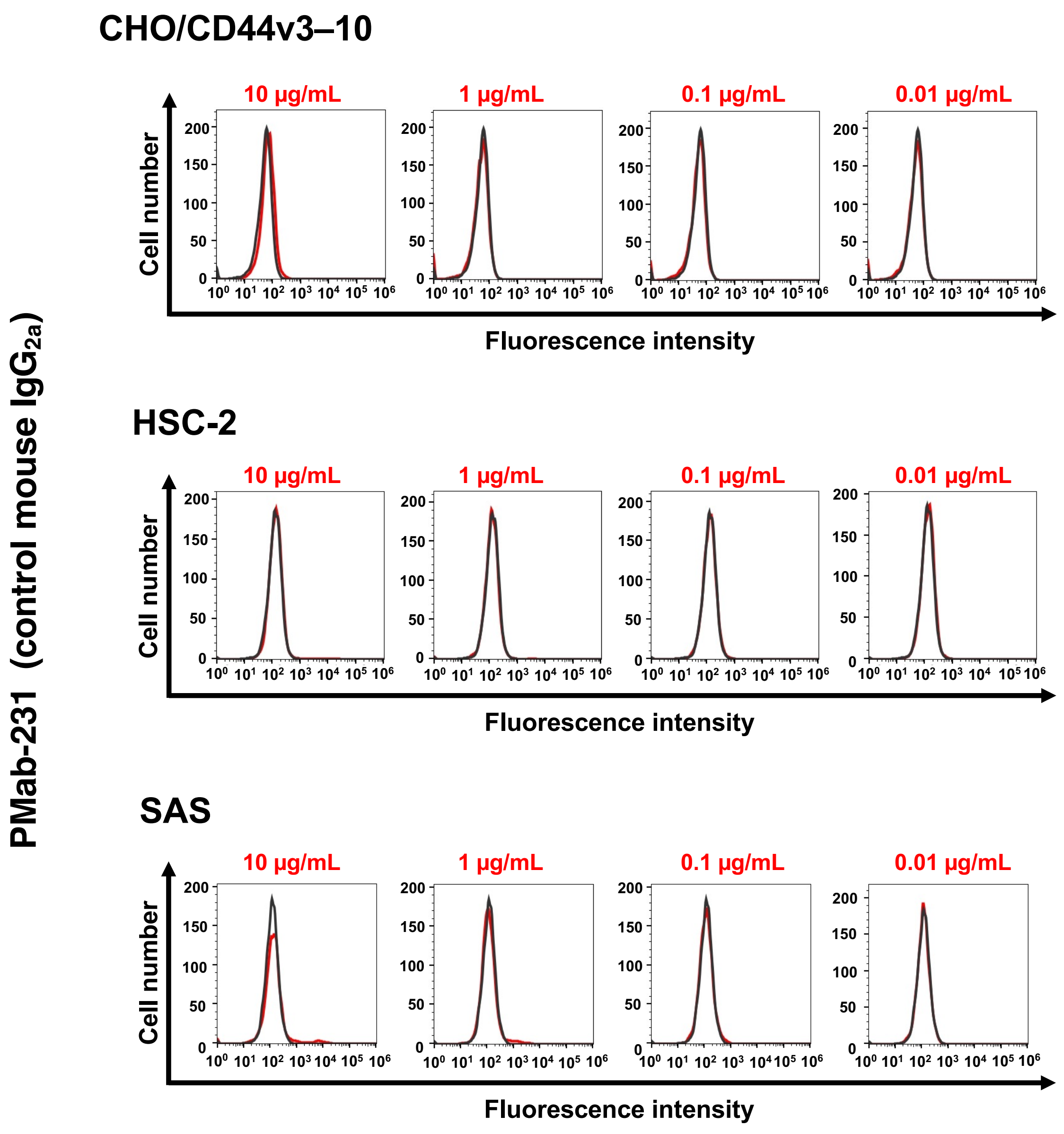

Supplementary Figure S3. Flow cytometry using control mouse IgG_{2a} (PMab-231) against CHO/CD44v3–10, HSC-2 and SAS cells. The cells were treated with buffer control (black) or 10-0.01 μg/mL of PMab-231 (red). The cells were further treated with Alexa Fluor 488-conjugated anti-mouse IgG. Fluorescence data were analyzed using the SA3800 Cell Analyzer.
